# The utility of dynamic MRI in differentiating the hormone-producing ability of pituitary adenomas

**DOI:** 10.1007/s11604-021-01121-9

**Published:** 2021-04-21

**Authors:** Taishi Amano, Tomohiko Masumoto, Hiroyoshi Akutsu, Noriaki Sakamoto, Sodai Hoshiai, Kensaku Mori, Takahito Nakajima

**Affiliations:** 1 Department of Diagnostic and Interventional Radiology, Faculty of Medicine, University of Tsukuba, 1-1-1 Tennodai, Tsukuba, Ibaraki 305-8575 Japan; 2 Department of Diagnostic Radiology, Toranomon Hospital, 2-2-2 Toranomon, Minato-ku, Tokyo, Japan; 3 Department of Neurosurgery, Faculty of Medicine, University of Tsukuba, 1-1-1 Tennodai, Tsukuba, Ibaraki 305-8575 Japan; 4 Department of Diagnostic Pathology, Faculty of Medicine, University of Tsukuba, 1-1-1 Tennodai, Tsukuba, Ibaraki 305-8575 Japan

**Keywords:** Magnetic resonance imaging, Dynamic contrast enhancement, Pituitary adenoma, Pituitary hormonal activity

## Abstract

**Purpose:**

To investigate the relationship between dynamic magnetic resonance imaging (MRI) findings and hormonal activity in pituitary adenomas.

**Methods:**

We retrospectively evaluated the dynamic MRI findings in 244 patients with pathologically confirmed pituitary adenomas and a diagnosis of clinically active prolactin (PRL)-producing adenomas. Among the 244 pituitary adenomas, 55, 16, 6, and 4 produced growth hormone (GH), PRL, adrenocorticotropic hormone, and thyroid-stimulating hormone, respectively, while 163 were non-functioning (NF) adenomas. For each adenoma, we calculated the washout rate (WR) and early (EER) and delayed (DER) tumour-to-normal-tissue enhancement ratios.

**Results:**

The respective mean values of the WR, EER, and DER were 9.4%, 75.2%, and 64.5% for GH-producing adenomas; 6.2%, 117.1%, and 106.2% for PRL-producing adenomas; and 5.4%, 116.7%, and 108.7% for NF adenomas. GH-producing adenomas had significantly lower EER and DER values than PRL-producing (*P* < 0.001) and NF adenomas (*P* < 0.001). In ROC analysis of GH-producing and non-GH-producing adenomas, the areas under the curves of WR, EER, and DER were 0.593, 0.825, and 0.857, respectively.

**Conclusion:**

There are differences in dynamic MRI features between GH-producing and non-GH-producing adenomas, which suggests that EER and DER may be useful for diagnosing GH-producing adenomas.

## Introduction

Pituitary adenomas are the most common intracranial endocrine tumours and possess various hormonal activities. Dynamic magnetic resonance imaging (MRI) facilitates the detection of pituitary microadenomas [1–5]; moreover, it is a useful diagnostic procedure for visualising pituitary glands displaced by large pituitary adenomas [1, 6]. Several studies have assessed the relationship between MRI features of pituitary adenomas and hormonal activity [7–9]; however, they mainly focused on non-contrast MRI findings. Guo et al. [10] performed a quantitative analysis on the contrast patterns of the dynamic MRI images of ACTH-producing pituitary adenomas and found that the tumour prepeak slope (referring to the measure of the tissue perfusion rate or speed reaching peak intensity in the enhancement curve) of ACTH-producing adenomas was significantly lower than that of non-functioning adenomas. In another report by Kanou et al. [11], which analysed the sequential MRI enhancement patterns of pituitary adenomas in 67 patients and the factors contributing to them, it was found that tumours with a very early enhancement pattern were significantly more fibrous than tumours with the other three patterns. However, no significant association between hormonal activity and MRI findings was reported. A recent study reported that dynamic MRI findings can predict the consistency of pituitary adenomas [12]; however, to our knowledge, there has been no statistical comparative analysis of hormonal activity and dynamic MRI findings of pituitary adenomas.

In our institution, all cases of pituitary adenomas, regardless of size, undergo dynamic MRI for preoperative evaluation. We have empirically found that dynamic contrast enhancement patterns of pituitary adenomas range from a greater washout pattern to a prolonged enhancement pattern. Dynamic MRI findings may be useful in predicting hormonal activity in case these enhancement patterns are associated with hormonal activity. This retrospective study aimed to assess the relationship between hormonal activity and dynamic contrast enhancement patterns of pituitary adenomas.

## Methods

### Patient population

This retrospective study was approved by the institutional review board, which waived the requirement for informed consent. We retrospectively studied MR images of pituitary adenomas obtained from 325 patients without previous surgical treatment for pituitary disease between August 2011 and February 2020. There were 163 and 162 male and female patients, respectively (age range: 8–90 years; mean age: 54 years).

### MRI parameters

MR images were acquired on a 1.5-T system (Achieva 1.5 T, Philips Healthcare, Best, the Netherlands), 3.0-T system (Achieva 3.0 T, Philips Healthcare, Best, the Netherlands), and 3.0-T system (Ingenia 3.0 T, Philips Healthcare, Best, the Netherlands). Imaging sequences included a sagittal and coronal T2-weighted fast spin-echo sequence (section thickness 3.0 mm; slice gap 0.3 mm; field of view [FOV] 160 mm; TE 80–90 ms; TR, 3000 ms; flip angle 90°). Dynamic MRI images were acquired using a sagittal and coronal T1-weighted fast spin-echo sequence (section thickness 3.0 mm; slice gap 0.3 mm; FOV 160 mm; TE 12 ms; TR 500–600 ms; flip angle 80°–90°). We administered a bolus injection of 0.1 mmol/kg of the contrast agent (gadoteridol; ProHance^®^, Bracco Eisai, Tokyo) at 2.5 mL/s, followed by 15 dynamic phases of MR images with a temporal sampling interval of 13 s and a total imaging time of 195 s.

Dynamic MR signal intensity values were measured in a circular or ovoid region of interest (ROI) area of between 0.15 and 2 cm^2^. Cystic, necrotic, or haemorrhagic regions in the pituitary adenoma were excluded from the ROI measurement. We defined the early phase as the timing of the maximum MR signal intensity values within three phases (39 s) after the signal increase in the pituitary stalk on T1-weighted images due to the passage of bolus contrast medium. Moreover, we defined the delayed phase as the last phase of fifteen dynamic phases (195 s). We obtained unenhanced MR signal intensity values for the pituitary tumour (Pre) and putamen (PT) for each patient. In addition, the signal intensities in the early phase (EP) and delayed phase (DP) on enhanced MR images were measured for pituitary tumours. The percentage washout of enhancement was defined as the washout rate (WR), which was calculated as follows: ${\text{WR}}\, = \,\left( {{\text{AP}} - {\text{DP}}} \right)/\left( {{\text{AP}} - {\text{Pre}}} \right)\, \times \,{1}00$. The tumour-to-putamen enhancement ratio for contrast effect in the early and delayed phases of a dynamic study of pituitary adenomas were defined as the early tumour-to-normal-tissue enhancement ratio (EER) and delayed tumour-to-normal-tissue enhancement ratio (DER), respectively $\left[ {{\text{EER}} = ({\text{AP}} - {\text{Pre}})/{\text{PT}} \times 100,\,{\text{DER}} = ({\text{DP}} - {\text{Pre}})/{\text{PT}} \times 100} \right]$ . Similarly, we obtained MR signal intensity values for the pituitary tumour and putamen from T2-weighted images. The T2 signal intensity ratio was calculated as follows: T2 signal intensity ratio = (intensity values of the tumour)/(intensity values of the putamen). The pituitary tumour size was measured as the maximum diameter (mm) on sagittal or coronal T2-weighted images.

### Statistical evaluation

The WR, EER, DER, T2 signal intensity ratio, and tumour size were analysed to determine their relationship with hormonal activity. Hormonal activity was classified as follows: growth hormone (GH)-producing adenomas, prolactin (PRL)-producing adenomas, non-functioning (NF) adenomas, and other adenomas (adrenocorticotropic hormone (ACTH) and thyroid-stimulating hormone (TSH)—producing adenomas). GH-producing adenomas were further classified into densely and sparsely granulated adenomas. Those variables were not normally distributed, and statistical analyses were performed using the Mann–Whitney *U* test. Statistical significance was set at a* P* value < 0.05. Bonferroni correction was applied for multiple comparisons. The receiver operating characteristic (ROC) curve was used to assess diagnostic performance.

## Results

**Fig. 1:**
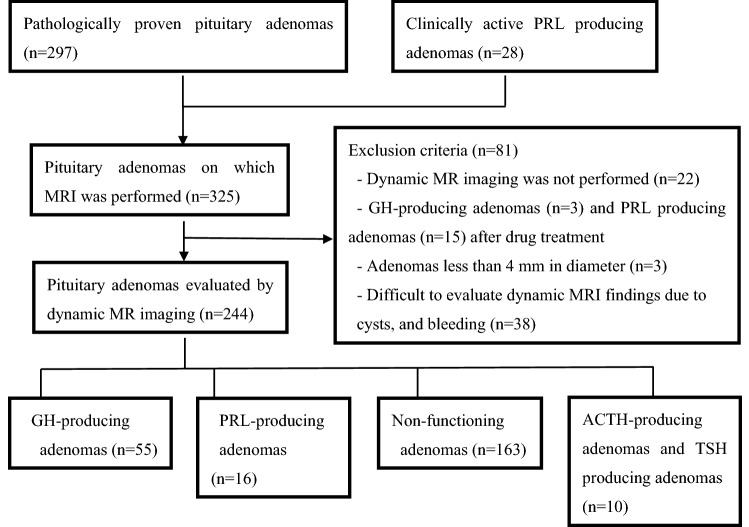
Flowchart of the patient enrolment process. (*PRL* prolactin, *GH* growth hormone, *ACTH* adrenocorticotropic hormone, *TSH* thyroid-stimulating hormone)

Figure 1 summarises the patient enrolment process. Among the 325 cases of pituitary adenomas, 297 adenomas were pathologically diagnosed at surgery, while 28 PRL-producing pituitary adenomas were diagnosed based on imaging studies and blood hormone levels. PRL-producing adenomas were classified through imaging of the pituitary adenoma and by the presence of serum PRL concentrations exceeding 200 ng/mL. PRL-producing adenomas were also classified through imaging of the pituitary microadenoma and by the presence of serum PRL concentrations between 50 and 200 ng/Ml, because NF macroadenomas may cause mild hyperprolactinemia due to pituitary stalk compression. We excluded 81 pituitary adenomas as follows: 22 adenomas that did not undergo dynamic MRI examinations; 3 GH-producing adenomas and 15 PRL-producing adenomas involving drug treatment before MRI examination; 3 small adenomas (< 5 mm), which impeded assessment through contrast-enhanced imaging; and 38 adenomas with severe cystic degeneration and haemorrhage, which impeded evaluation of contrast images. Among the remaining 244 cases of pituitary adenomas, 55, 16, 6, and 4 produced GH, PRL, ACTH, and TSH, respectively, while the remaining 163 were clinically diagnosed as NF adenomas. The latter lacked pituitary hormone hypersecretion and included multiple histological types, such as silent histology gonadotroph adenomas, silent corticotroph adenomas, and null cell adenomas. Among the 55 GH-producing adenomas, 43 were subclassified pathologically as either densely or sparsely granulated. Of the remaining 12 cases, 3 were subclassified as mixed, while 9 were unclassifiable.

**Fig. 2:**
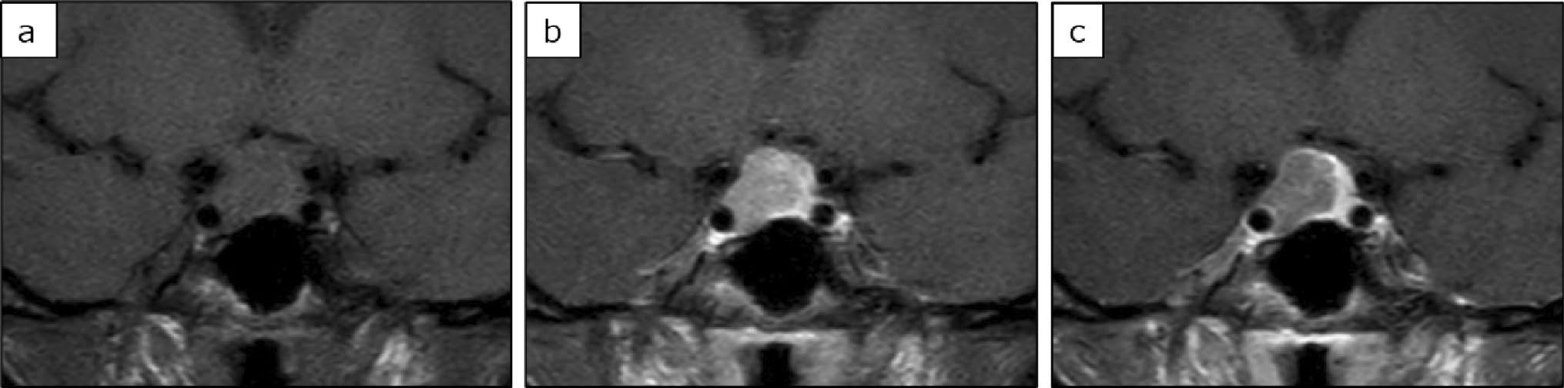
A 44-year-old woman with growth hormone producing pituitary adenoma. The tumour was isointense to the brain on unenhanced T1 weighted imaging (**a**); moreover, it was weakly contrasted in the early phase (**b**) and washed out in the delayed phase (**c**). The WR was relatively high (44.5%), EER was relatively low (86.6%), and DER was relatively low (48%), which is a common pattern in GH adenomas

**Fig. 3:**
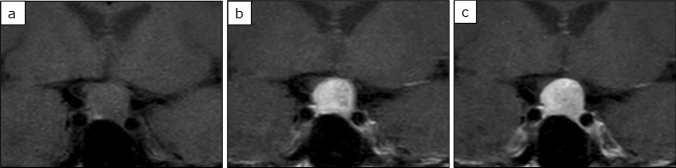
A 64-year-old woman with non-functioning pituitary adenoma. The tumour was isointense to the brain on unenhanced T1 weighted imaging (**a**); moreover, it was strongly contrasted in the early phase (**b**) while the contrast effect was prolonged in the delayed phase (**c**). The WR was relatively low (2.2%), EER was relatively high (145.5%), and DER was relatively high (142.2%), which is a common pattern in non-GH adenomas

**Fig. 4:**
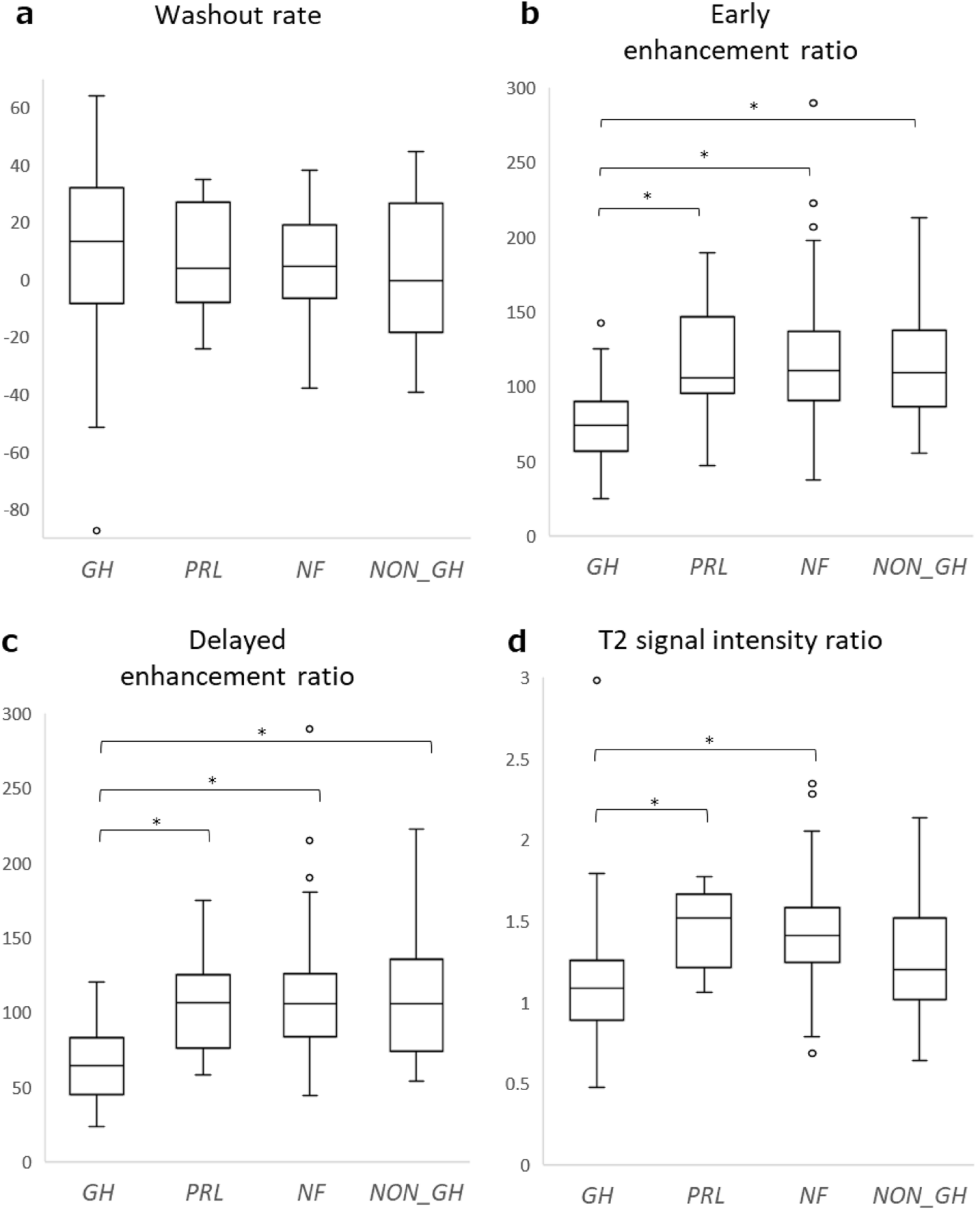
Comparison of WR, EER, DER, and T2 signal intensity ratio for each hormone producing adenomas. (*GH* GH producing adenoma, *PRL* PRL producing adenoma, *NF* NF adenoma, *OTHERS* ATCH and TSH producing adenoma). The Bonferroni correction was applied for multiple comparisons. A *P* value < 0.0125 was considered significant for this analysis. **a** Box and whisker plots showed that the WR tended to be higher in *GH* than in *NF*; however, the difference was not statistically significant (*P* = 0.039). **b** The EER was significantly lower in *GH* than in *PRL*, *NF*, and *OTHERS* (*P* < 0.001, *P* < 0.001, and *P* = 0.004, respectively). **c** The DER was significantly lower in *GH* than in *PRL*, *NF*, and *OTHERS* (*P* < 0.001, *P* < 0.001, and *P* = 0.001, respectively). **d** T2 signal intensity ratio was significantly lower in *GH* than in *PRL* and *NF* (*P* < 0.001 and *P* < 0.001, respectively)

**Fig. 5:**
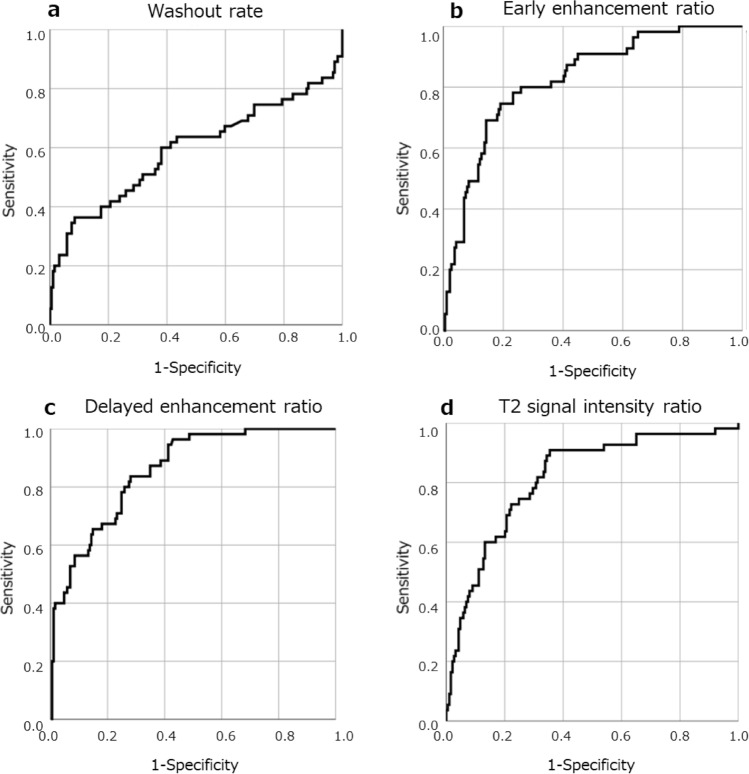
ROC curve of the WR, EER, DER, and T2 signal intensity ratio for predicting GH-producing pituitary adenoma. The area under the ROC curves for WR (**a**), EER (**b**), DER (**c**), and T2 signal intensity ratio (**d**) were 0.593, 0.825,0.857, and 0.814 respectively

Table 1 summarises the MRI findings. The respective mean values of WR, EER, and DER were 9.4%, 75.2%, and 64.5% for GH-producing adenomas; 6.2%, 117.1%, and 106.2% for PRL-producing adenomas; and 5.4%, 116.7%, and 108.7% for NF adenomas. Dynamic MR images indicated that compared with other adenomas, GH-producing adenomas tended to have a higher WR and a lower EER and DER. A typical example is shown in Fig. 2. Non-GH-producing tumours, including NF adenomas, tended to have a lower WR and a higher EER and DER. A typical example is shown in Fig. 3. The WR was non-significantly higher in GH-producing adenomas than in NF adenomas (*P* = 0.039) (Fig. 4a). The EER was significantly lower in GH-producing adenomas than in PRL-producing adenomas, NF adenomas, and other adenomas (*P* < 0.001, *P* < 0.001, and *P* = 0.004, respectively) (Fig. 4b). Moreover, DER was significantly lower in GH-producing adenomas than in PRL-producing adenomas, NF adenomas, and other adenomas (*P* < 0.001, *P* < 0.001, and *P* = 0.001, respectively) (Fig. 4c). ROC curves focusing on the differential diagnosis between GH- and non-GH-producing adenomas were analysed using data from WR, EER, and DER. The areas under the curves for the WR, EER, and DER were 0.593, 0.825, and 0.857, respectively (Fig. 5).

**Table 1:** MR imaging findings of pituitary adenomas

Adenoma type	Number of cases	Sex (m/f)	Age	T2 signal intensity ratio	Maximum diameter (mm)	WR (%)	EER (%)	DER (%)
All types	244	133/111	55 (± 14)	1.34 (± 0.33)	26.8 (± 1.1)	6.2 (± 21.4)	107.3 (± 38.8)	98.7 (± 37.7)
GH producing	55	22/33	50 (± 13)	1.09 (± 0.37)	20.2 (± 9.1)	9.4 (± 31.4)	75.2 (± 25.6)	64.5 (± 22.7)
Densely granulated	31	12/19	53 (± 12)	1.06 (± 0.41)	18.3 (± 9.1)	11.0 (± 26.8)	73.9 (± 27.1)	62.6 (± 21.7)
Sparsely granulated	12	5/7	40 (± 10)	1.17 (± 0.33)	25.8 (± 6.4)	9.7 (± 40.0)	84.0 (± 29.4)	69.9 (± 26.3)
non-GH producing	189	103/86	57 (± 14)	1.41 (± 0.29)	28.8 (± 10.9)	5.3 (± 17.4)	116.0 (± 38.0)	108 (± 36.5)
PRL producing	16	8/8	40 (± 14)	1.14 (± 0.22)	22.7 (± 11.5)	6.2 (± 19.1)	117.1 (± 35.1)	106.2 (± 35.1)
Non-functioning	163	93/70	59 (± 13)	1.45 (± 0.27)	30.3 (± 10.2)	5.4 (± 16.7)	116.7 (± 36.6)	108.7 (± 35.1)
ACTH producing	6	0/6	47 (± 20)	1.02 (± 0.22)	12.2 (± 5.4)	1.9 (± 25.8)	94.6 (± 32.2)	89.2 (± 27.7)
TSH producing	4	2/2	52 (± 12)	1.68 (± 0.37)	16.5 (± 8.1)	1.7 (± 32.4)	147.1 (± 46.7)	142.5 (± 60.1)

Values are averages and data in parentheses are standard deviations

On T2-weighted MR images, GH-producing adenomas had lower signal intensities than non-GH-producing adenomas. The T2 signal intensity ratio was significantly lower among GH-producing adenomas than among NF adenomas (*P* < 0.001); and was also significantly lower in GH-producing adenomas compared to PRL-producing adenomas (*P* < 0.001) (Fig. 4d). ROC curves between GH- and non-GH-producing adenomas revealed that the area under the curve of T2 signal intensity ratio was 0.814 (Fig. 5d). There were no significant differences in T2 signal intensity ratio between GH-producing adenomas and other adenomas. Densely granulated GH-producing adenomas had a significantly lower T2 signal intensity ratio than sparsely granulated adenomas (*P* = 0.04). Contrastingly, there was no significant difference in WR, EER, and DER between densely and sparsely granulated GH-producing adenomas.

GH-producing adenomas had significantly smaller tumour sizes than non-GH-producing adenomas (*P* < 0.001). The correlation coefficients between tumour size and dynamic MRI features (WR, EER, and DER) were 0.030, 0.090, and 0.079, respectively. Cystic, necrotic, or haemorrhagic regions were present in 120 of the 244 adenomas.

## Discussion

Our findings indicate that dynamic MRI could be used to predict GH-producing adenomas in pituitary adenomas. Compared with non-GH-producing adenomas, GH-producing adenomas showed a weaker enhancement pattern on the early and delayed phases. ROC analysis for differentiating GH-producing from non-GH-producing adenomas showed high AUC values of 0.825 and 0.857 for EER and DER, respectively, with a specifically high diagnostic performance for DER. Consistent with the findings by Hagiwara [9], we found that GH-producing adenomas had a lower T2 signal intensity ratio than PRL-producing adenomas and NF adenomas. However, there was no significant difference in the T2 signal intensity ratio for other adenomas. These findings suggest that dynamic MRI could allow differentiation of GH adenomas from other adenomas, which was not adequately allowed by previous T2WI findings. Furthermore, the AUC value of the T2 signal intensity ratio was 0.814, indicating that the AUC value (0.857) for DER was higher than that of the T2 signal intensity ratio.

Surgery for GH-producing adenomas requires more complete tumour removal than surgery for non-functioning adenomas; moreover, preoperative diagnosis is important. In most cases, the diagnosis of acromegaly can be made by observing clinical symptoms and the presence of GH or Insulin-like growth factor-1 (IGF-1) in the blood. However, in some cases, clinical symptoms are insufficient, and diagnosis is difficult. On the contrary, some cases of low GH acromegaly show low GH levels in the blood but present clinical symptoms, whereas IGF-1 levels in the blood may be low in those with a low nutritional status, liver disorders, and poorly controlled diabetes mellitus. Our MRI findings may be useful for clinical diagnosis in such cases. Moreover, it may be useful to differentiate GH-producing adenomas by imaging diagnosis in cases of pituitary apoplexy or severe visual dysfunction that require urgent or quasi-urgent surgery, because the IGF-1 blood test is often outsourced and generally has a long turn-around time.

In this study, GH-producing adenomas were smaller than NF adenomas. Given that the tumour size might be associated with dynamic MRI findings, we analysed the correlation between tumour size and dynamic MRI features (WR, EER, and DER). None of the variables were found to be closely correlated with tumour size. This suggests that the pattern differences across the adenomas were not associated with tumour size; however, they could be attributed to differences in other histological structural features.

Vidal et al. [13] reported that GH-producing adenomas had a significantly lower microvascular density than PRL-producing and NF adenomas, which is consistent with the lower EER and DER for GH-producing adenomas than for other adenomas. Di et al. [14] reported that functional adenomas showed a non-significantly lower microvascular surface area than non-functional adenomas. Similarly, this is consistent with our finding that GH-producing adenomas had lower EER and DER values than the NF adenomas. Takano et al. [15] reported that GH-producing macroadenomas tended to have significantly and non-significantly smaller vessel diameters and circumferences than PRL-producing macroadenomas and NF macroadenomas, respectively. This is also consistent with the lower EER and DER in GH-producing adenomas than in PRL-producing adenomas and NF adenomas.

Densely granulated GH-producing adenomas are often effective with somatostatin analogues and have a lower signal on T2WI [9], which is consistent with the lower T2 signal intensity ratio of densely granulated adenomas than in sparsely granulated adenomas. Wei et al. [16] reported that pituitary adenomas with a lower T2WI signal ratio of the tumour and pons had a higher collagen content. Moreover, differences in dynamic MRI contrast patterns were expected to result from histological structural differences. However, we found no difference in the WR, EER, and DER between densely and sparsely granulated types.

Dynamic MRI is useful for detecting pituitary microadenomas, which are difficult to identify on non-contrast MRI, since the normal pituitary gland is very strongly contrast-enhanced, while adenomas show a relatively low intensity. However, we found that GH-producing adenomas had lower contrast enhancement than PRL-, ACTH-, and TSH-producing adenomas. This suggests that the latter adenomas could yield inferior contrast between the adenoma and normal pituitary gland compared with GH-producing adenomas when examining for microadenomas on dynamic MRI. Moreover, ROC analysis for differentiating between GH-producing and non-GH-producing adenomas showed high diagnostic performance, especially for DER. This suggests that it may be better to focus on the delayed phase, rather than the early phase when detecting and diagnosing GH-producing adenomas on dynamic MRI.

### Limitations

The main limitation of this study is the mixed use of different imaging MRI systems. In addition, this study had a limited number of cases of PRL-producing adenomas due to many patients being on drug treatment at the time of referral to our hospital. Moreover, patients who were controllable through medical treatment did not undergo preoperative MRI, including dynamic study, at our institution.

## Conclusion

Dynamic MRI could be used to predict GH-producing adenomas in pituitary adenomas. Compared with NF adenomas, PRL-producing adenomas, and other adenomas, GH-producing adenomas had a weaker enhancement pattern on the early and delayed phases. In addition, compared with GH-producing adenomas, PRL-, ACTH-, and TSH-producing adenomas could yield inferior contrast between the adenoma and normal pituitary gland when searching for microadenomas on dynamic MRI. It may be better to focus on the delayed phase, rather than the early phase for detecting and diagnosing GH-producing adenomas on dynamic MRI.
